# Parents’ screen time, parental perception, technology-related parenting in relation to young children’s screen time: a cross-sectional study

**DOI:** 10.1186/s40359-025-03574-3

**Published:** 2025-12-18

**Authors:** Drishti Bhoi, Jeeshma Vijin, HA Venkatesh

**Affiliations:** 1 Department of Clinical Psychology, Manipal College of Health Professions, Manipal Academy of Higher Education, Manipal, India; 2 Department of pediatrics, Manipal Hospital, old airport road, Bengaluru, India

**Keywords:** Young children, Screen time, Parental perception, Technology-related parenting

## Abstract

**Background:**

Excessive screen time in children has become an increasing concern as it leads to various developmental delays, including negative psychological outcomes and impaired cognitive and socio-emotional development. Existing literature suggests that parental behavior may influence children’s screen use, as young children often model parental habits. Given the rising prevalence of screen exposure in this age group, it is critical to investigate the factors contributing to increased screen time, particularly in the Indian context.

**Methods:**

The current cross-sectional study was conducted on parents (16–40 years) and their young children (2–6 years) from Manipal Hospital, Old Airport Road, Bangalore. Participants were recruited using a convenience sampling method. The Screen Time Questionnaire assessed demographic details and children’s screen time. Parents’ screen time was measured using the Instrument for the Assessment of Internet Use. The Technology-Related Parenting Scale evaluated parental behavioral strategies for managing child technology use, while the Parental Perceptions of Technology Scale measured negative attitudes and perceived parental efficacy. A Shapiro-Wilk test for normality was conducted, and subsequently, Spearman’s Rho correlation was used to understand the relationship between the screen time of parents and children.

**Results:**

The current study revealed that there is a weak and statistically non-significant positive relationship between the average screen time of the child and parents (ρ = 0.103, *n* = 123, *p* = .256), indicating a weak relation between them. Similarly, children’s screen time was not significantly associated with negative attitudes of parents (ρ = 0.039, *n* = 123, *p* = .671) and parental efficacy (ρ = -0.018, *n* = 123, *p* = .845). A weak but statistically significant negative correlation was observed between children’s screen time and technology-related parenting (ρ = -0.222, *n* = 123, *p* = .014), suggesting that the implementation of rules and monitoring screen time of children is associated with lower screen time among children.

**Conclusion:**

The findings suggest that there is no significant association between children’s and their parents’ screen time or parental perceptions of technology. However, technology-related parenting demonstrates a weak but significant negative association with children’s screen time.

**Trial registration:**

The study was registered in the clinical trial registry in India (CTRI). CTRI registration CTRI/2025/03/081956 http//ctri.nic.in/ [Registered on 08/03/2025].

## Background

Screen time, defined as the time spent using electronic devices such as televisions, computers, mobile phones, tablets, and video game consoles [1], has become a major concern due to its detrimental effects on early childhood development amid rapid technological advancement. Organizations like the American Academy of Pediatrics (AAP) and the World Health Organization (WHO) recommend no screen exposure for infants (under 18 months or under 1 year, respectively), except for supervised video calls. For children aged 18–24 months, the AAP advises only high-quality, co-viewed content. By ages 2–5 years, or 2–4 years, as per WHO, screen time should be limited to one hour per day, interactive, and involve active parental supervision [2]. It is further advised against having screen time during meals and before bedtime and emphasizes not using screens as babysitters or pacifiers [2].

Despite these guidelines, ownership of digital devices has surged, and preschoolers increasingly exceed recommended limits [3, 4]. Studies in India have shown that preschoolers have an average of 2.7 h of screen time daily, which is more than the recommended amount [1, 5, 6]. An increasing number of studies have found that excessive screen exposure in early childhood is linked to language delays, impaired cognitive and socio-emotional development, reduced white matter integrity, and symptoms resembling ADHD or autism spectrum disorder [5, 7–10]. Additional risks include impulsivity, inattention, emotional dysregulation, aggressive behavior, weak caregiver-child bonds, poor academic performance, and hindered social competence [11–14]. Higher screen time is also associated with increased reward orientation and weaker fronto-striatal connectivity, affecting inhibitory control, a critical function for regulating attention, emotions, and behavior [15]. Moreover, children and adolescents who spend five or more hours daily on screens have over twice the risk of vitamin D deficiency compared to those with less screen time, emphasizing the need to evaluate screen habits in pediatric care, as excessive screen use can limit outdoor activity and sunlight exposure [16].

Parental influence emerges repeatedly as a key determinant of children’s screen habits in various studies. Studies indicate that parents’ own screen time behaviors, attitudes toward technology, self-efficacy in enforcing limits, and rule setting are all correlated with their children’s screen time [17–20]. Similarly, theories such as Bandura’s social learning and Bronfenbrenner’s ecological systems model explain how young children emulate parental media use, particularly when unsupervised [17]. Likewise, previous studies have identified maternal screen use before bedtime, absence of household rules, and positive parental attitudes toward screen use as contributing to increased child screen exposure, whereas clear screen-time rules and parental monitoring are associated with reduced usage [5, 19–22]. Additionally, socio-demographic factors such as family income, parental education, and device access also shape children’s media patterns [23–25].

Previous research has focused primarily on school-going children or adolescents, with limited research addressing younger children [21, 26]. Few studies investigate parental screen time alongside perceptions and technology-related parenting. Technology-related parenting is how parents manage and control their children’s use of technology, including setting rules about screen time and content access and enforcing consequences to promote healthy and safe technology use [27]. In India, cultural norms, family dynamics, and technology access differ notably from Western contexts [6, 21, 25, 26, 28]. In India, early screen exposure in joint families, limited parental control due to working caregivers, and widespread device access shape children’s screen time. In contrast, studies from Australia and Europe were found to report lower screen time and stricter parental regulation [5]. There is also increased availability and affordability of digital devices across different socioeconomic groups, alongside decreased traditional outdoor play and social interaction in India, which accentuates a critical research gap. This highlights the need for studies exploring screen time patterns, parental attitudes, and technology-related parenting in India to inform culturally relevant guidelines and interventions.

The present study addresses these gaps by examining the association between parents’ screen time and their children’s screen time, the relationship between parental perceptions of technology and children’s screen time, and the impact of technology-related parenting on young children’s screen exposure in an Indian context.

## Methods

### Study design and settings

The aim of this research was to investigate the relationship between parents’ screen time and that of their young children. The study employed a correlational design, and data were collected using convenience sampling from parents coming to the Manipal Hospital Pediatric Department, HAL, Bengaluru. Convenience sampling was selected due to its practical feasibility, as it allowed easy access to the target population within the available time and resources.

###  Study procedure

Parents aged 16–40 years, as the scale used to assess screen time of parents is validated on participants ranging from 16 to 40 years with children aged 2–6 years, were eligible for the study if they reported at least 2 h of daily screen time. Parents were eligible if they reported at least 2 h of daily screen time, excluding work-related use. This threshold was chosen because the study focused on parental modeling of screen habits, under the assumption that greater parental use increases opportunities for children to observe and potentially adopt these behaviors. Screen time on devices such as mobile phones, laptops, computers, televisions, and tablets was considered. Both mothers and fathers were included. Screen time related to parents’ work hours, whether at an office or while working from home, was excluded; parents were asked to report only their leisure-related screen use. Data were collected from the parent who reported spending more screen time in the presence of the child, as this parent was considered the primary model of the child’s screen use behavior. In cases where only one parent was present at the outpatient department (OPD) during data collection, data were collected solely from that parent. For households with multiple children, data on screen time, parental rules, and attitudes were collected for each child.

The sample size (N) for this study was calculated using the formula N = [(Zα + Zβ)/C(r)]² + 3, where *Zα* corresponds to the standard normal deviate at a significance level of 0.05 (95% confidence interval = 1.96), *Zβ* corresponds to the standard normal deviate at 80% power (0.20 = 1.64), and *C(r)* represents the Fisher’s Z transformation of the expected correlation coefficient (*r*). With an anticipated correlation strength of *r* =.7, the calculation yielded a required sample size of approximately 29–30 participants per variable. Since the study examined four variables, the total estimated sample size was 120. To ensure adequate representation, a total of 123 parent-child pairs were ultimately included in the study.

The study procedures were implemented according to the guidelines of the institutional ethical committee and aligned with the ethical standards of the Declaration of Helsinki. Informed consent was obtained from all the parents.

## Measures

### Screen time questionnaire [4]

The Screen Time Questionnaire, directly adapted from John et al. (2021), was used to collect demographic and screen-related data for children. This questionnaire gathered information on the child’s age and gender, parental education and employment status, frequency and duration of the child’s screen time at home, types of devices used, and the child’s involvement in extracurricular activities. Participants reporting on this questionnaire were the parents, providing reports of their children’s screen habits. The questionnaire was adapted specifically for this study context based on the referenced research, enabling culturally relevant assessment of screen-time variables.

### Instrument for the assessment of internet use [23]

Instrument for the Assessment of Internet Use was developed by Palanichamy et al. for an Indian population ranging from 16 to 40 years; this 18-item scale measures device use across three domains. First is recreational use (score range 0–18), second is excessive use (19–36), and lastly is dysfunctional use (37–54) of the Internet. Each item is rated on a 4‐point Likert scale (0 = never, 1 = sometimes, 2 = often, 4 = always). This instrument demonstrated satisfactory psychometric properties of reliability, with a value of 0.893, and high content validity. Parents self-reported their screen time while excluding work-related screen use.

### The technology-related parenting scale [27]

The Technology-Related Parenting Scale was developed by Sanders et al. (2016); this 8-item scale assesses behavioral management strategies that parents employ to regulate their child’s technology use. Items are rated on a 3-point Likert scale (0 = not true, 1 = somewhat true, 2 = very true), with higher total scores indicating more extensive rule‐setting and enforcement around screen time with 16 being the highest score. This measure has demonstrated good reliability and concurrent validity in previous research. Parents reported their own behaviors regarding screen use rules and supervision.

### Parental perceptions of technology scale [27]

Also developed by Sanders et al. (2016), the Parental Perceptions of Technology Scale is a 10-item scale that evaluates two dimensions: first is Negative Attitudes toward technology (4 items), and second is Perceived Parental Efficacy (6 items). Items were rated on a 5-point Likert scale (1 = strongly disagree to 5 = strongly agree). Higher scores on each subscale reflected stronger negative beliefs (maximum score = 20) or greater parental self-efficacy (maximum score = 30).

Satisfactory reliabilities were found: 0.72 (Negative Attitudes) and 0.83 (Perceived Efficacy). This scale was used to understand parental attitudes and perceived competence related to technology. Parents provided self-reports of their attitudes and perceived efficacy.

None of the scales was specifically adapted for this study. However, all were selected due to their satisfactory psychometric properties and were used with permission from the respective authors. The Screen Time Questionnaire [4] and the Instrument for the Assessment of Internet Use [23] were originally developed and validated for Indian populations, ensuring cultural relevance. In contrast, the Technology-Related Parenting Scale [27] and the Parental Perceptions of Technology Scale [27] were developed in non-Indian contexts and have not been formally validated within the Indian population, representing a limitation of this study.

### Data analysis

Jamovi ver. 2.6.26 [29] was used for data analysis. Demographic characteristics were analyzed using descriptive statistical methods such as mean and standard deviation. For the scored data, Shapiro-Wilk’s test of normality was conducted to determine if the data were normally distributed. Since the data was not normally distributed, a Spearman’s Rho Correlation test was conducted.

## Results

A total of 123 participants were included in the study. Socio-demographic characteristics of the participants and related behavioral details are summarized in Tables 1 and 2. The mean age of the children was 3.85 years (SD = 1.05). On average, children spent 1.80 h per day (SD **=** 0.95) on screen-based activities. The mean duration of daily physical activity among children was 2.61 h (SD = 1.26). Parents reported a mean daily screen time of 1.96 h (SD = 1.25). The average age of the parents was 34.25 years (SD = 3.50).

There were slightly more female children (51.2%) than boys, and a majority were attending school (71.5%). Parents were the primary caretakers in most cases, though in about one-third of households, childcare was shared with grandparents. Regarding screen-related practices, digital devices were most commonly introduced between 1 and 3 years of age (51.2%). Screen time was supervised all the time in 62.6% of children. Parental monitoring of content was high (90.2%), and co-viewing was reported at least some of the time by 82.9% of parents. Only a small proportion of children (9.8%) owned personal devices. Most parents reported establishing screen time limits (65.0%) and screen-free intervals (55.3%). Notably, nearly half the children (48.4%) were exposed to screens one hour before their bedtime. With regard to behavioral outcomes, speech delay was reported in 5.7% of children. 17.1% of parents expressed difficulty in controlling their child’s screen use. Screen use during meals was common, with 48.0% reporting device use at all mealtimes. Tantrums related to screen time occurred in 56.9% of children. Most parent participants were mothers (77.2%), with fathers comprising 22.8% of respondents.

The majority of mothers were graduates (78.9%), with a smaller proportion attaining postgraduate education (21.1%). Most mothers were working (74.8%) rather than housewives (25.2%). Fathers had varied educational backgrounds, with 67.5% holding a graduate degree, 21.1% being postgraduates, and smaller shares completing 12th grade (6.5%) or 10th grade (4.9%). Occupationally, fathers worked mostly as engineers (21.1%), in business (11.4%), in healthcare (8.9%), or in other employed roles (56.9%), with a small minority unemployed (1.6%).

Screen use among parents was primarily normal 82.1%, with 17.9% reporting excessive use. Negative parental attitudes toward screen time had a mean score of 13.2 (SD 4.4), indicating a moderate negative attitude. Perceived parental efficacy related to screen time was low (mean 14.5, SD 4.9). In contrast, technology-related parenting that regulate screen use averaged 10.1 (SD 3.2), reflecting moderate rule-setting around screen exposure.

**Table 1:** Socio-demographic characteristics

	Mean	SD
Age of the child (years)	3.85	1.05
Average screen time of the child (hours/day)	1.80	0.95
Devices used by the child (count)	3.69	2.94
Physical activity by children (hours/day)	2.61	1.26
Screen time of parents (hours/day)	1.96	1.25
Age of parents	34.25	3.50
Parent’s perceptions of technology (Attitudes)	13.65	4.52
The perceived parental efficacy	14.93	4.70
Technology-related parenting	9.78	3.48

*SD* = Standard deviation

**Table 2:** Frequency of socio demographic characteristics

Demographics		Counts	% of Total
Gender of the child	Male	60	48.8%
	Female	63	51.2%
School	Doesn’t go to school	35	28.5%
	Goes to school	88	71.5%
Caretaker of the child	Parents	45	36.6%
	Parents, Grandparents	35	28.5%
	Grandparents	20	16.3%
	Parents, Others	9	7.3%
	Parents, Maid	6	4.9%
	Parents, Grandparents, Maid	2	1.6%
	Parents, Grandparents, Others	3	2.4%
	Others	1	0.8%
	Maid	2	1.6%
Supervision of screen time	Never	10	8.1%
	Some of the time	36	29.3%
	All of the time	77	62.6%
Age at which devices were introduced	Between 0–12 months	34	27.6%
	Between 1–3 years	63	51.2%
	Between 3–5 years	26	21.1%
Context in which child uses screen time	On demand	45	36.6%
	As a Reward	18	14.6%
	To pacify the child in stressful conditions	8	6.5%
	On demand, As a Reward	4	3.3%
	On demand, To pacify the child in stressful conditions	11	8.9%
	As a Reward, To pacify the child in stressful conditions	4	3.3%
	On demand, As a Reward, To pacify the child in stressful conditions	10	8.1%
	Meal time	13	10.6%
	None	5	4.1%
	As a Reward, To pacify the child in stressful conditions, Meals	2	1.6%
	On demand, Meal time	3	2.4%
Content monitoring by parents	No	12	9.8%
	Yes	111	90.2%
Coviewing with children	All the time	46	37.4%
	Some of the time	56	45.5%
	Never	21	17.1%
Children having own devices	No	111	90.2%
	Yes	12	9.8%
Screen time limits established by parents	No	43	35.0%
	Yes	80	65.0%
Screen free intervals established by parents	No	55	44.7%
	Yes	68	55.3%
Child interacting with peers	Yes	3	2.4%
	No	120	97.6%
Screen time before bedtime	Child goes off to sleep while watching the digital device	12	9.8%
	1 h	59	48.4%
	2 h	20	16.4%
	3 h	8	6.6%
	More than 3 h	23	18.9%
Speech delay in children	No delay	116	94.3%
	Delay	7	5.7%
Parents being able to control child’s screen time	No	21	17.1%
	Yes	102	82.9%
Device use during meal time	Never	27	22.0%
	Some of the time	37	30.1%
	All of the time	59	48.0%
Tantrums thrown for screen time	Never	32	26.0%
	Some of the time	70	56.9%
	All the time	21	17.1%
Gender of parents	Father	28	22.8%
	Mother	95	77.2%
Age of parents	26–30	38	30.89%
	31–35	43	34.96%
	36–40	42	34.15%
Level of education of mothers	Graduates	97	78.86%
	Postgraduates	26	21.14%
Level of education of fathers	Postgraduates	26	21.14%
	Graduates	83	67.48%
	12th grade	8	6.50%
	10th grade	6	4.88%
Mother’s Occupation	Housewives	31	25.20%
	Working	92	74.80%.
Father’s Occupation	Engineers:	26	21.14%
	Business	14	11.38%
	Healthcare	11	8.94%
	Employed (other)	70	56.91%
	Unemployed	2	1.63%
Screen use of parents	Recreational use	101	82.1%
	Excessive use	22	17.9%

Test for normality and Spearman’s correlation coefficients for primary variables are presented in Tables 3 and 4. Normality testing for 123 participants using the Shapiro-Wilk test (see Table 3) indicated that most variables were not normally distributed (*p* <.05), supporting the use of non-parametric methods.

**Table 3:** Test for normality

	Shapiro-Wilk	
	W	*p*
Screen time of parents	0.433	< 0.001
Parent’s perceptions of technology (attitudes)	0.941	< 0.001
The Perceived Parental Efficacy	0.964	0.002
Technology related parenting	0.946	< 0.001

**Table 4:** Correlational matrix

		Screen time of children	Screen time of parents	Parent’s perceptions of technology (attitudes)	The Perceived parental efficacy	Technology related parenting
Screen time of children	Spearman’s rho	—				
	df	—				
	*p*-value	—				
Screen time of parents	Spearman’s rho	0.103	—			
	df	121	—			
	*p*-value	0.256	—			
Parent’s perceptions of technology (attitudes)	Spearman’s rho	0.039	0.089	—		
	df	121	121	—		
	*p*-value	0.671	0.326	—		
The Perceived parental efficacy	Spearman’s rho	−0.018	−0.004	0.314***	—	
	df	121	121	121	—	
	*p*-value	0.845	0.961	< 0.001	—	
Technology related parenting	Spearman’s rho	−0.222*	−0.083	0.209*	0.133	—
	df	121	121	121	121	—
	*p*-value	0.014	0.363	0.021	0.142	—

*p* <.05, ** *p* <.01, *** *p* <.001

Correlation analysis (Table 4) indicated that child screen time was not significantly associated with parental screen time, parental attitudes, or perceived efficacy. However, a significant negative correlation was observed with technology-related parenting (ρ = − 0.222, *p* =.014), higher scores on technology-related parenting (indicating stricter rules) are associated with lower child screen time.

## Discussion

The present study aimed to examine the relationship between screen time of parents, parental perceptions of screen time, technology-related parenting, and screen time of children. Overall, the study found weak, statistically non-significant associations between parents’ screen time, parental attitudes/self-efficacy, and children’s screen use, while technology-related parenting was more strongly associated. These findings highlight the complex and context-dependent nature of children’s screen exposure.

The very weak correlation between parents’ and children’s screen time, indicating that parental screen time did not influence children’s screen time, does not align with the existing literature, which has often suggested that children model parental behavior in this domain.

One possible explanation is that many parents in the study reported using devices during times when children were not present, for instance, after the child had gone to sleep, thereby minimizing the opportunity for observational learning. This lack of visibility could weaken the modeling effect. Furthermore, co-viewing complicates the interpretation of associations between parent and child screen time. When parents engage in joint screen time with their children, their child’s reported screen time may be elevated without necessarily reflecting independent use. Evidence suggests that co-viewing is not a uniform practice. When parents co-view intentionally by monitoring content, modeling balanced use, or scaffolding self-regulation, it may support healthy engagement. In contrast, incidental co-viewing may normalize higher screen exposure. These differences help explain mixed findings in the literature and highlight the importance of considering parental intentions. Clarifying how co-viewing is measured is therefore essential for accurate interpretation and should be addressed in future research [30].

Additionally, contextual stressors and parenting strategies may play a critical role. Parents often resorted to giving devices to children to pacify them or to manage behavioral issues, rather than because of children observing their own screen use. Prior research supports this, noting that parents frequently use screens as “babysitters” to manage tantrums or to buy time for themselves, particularly in households where both the parents are employed. Family stress and reduced parental capacity for active engagement have been linked to greater reliance on screen-based “babysitting.” Research highlights that heightened stress or fatigue can reduce parents’ adherence to screen time guidelines, leading to more permissive practices [19]. This may be partly because parents who are under stress do not have access to other coping mechanisms including expressing emotions, taking a break, meditating, playing outside, laughing, concentrating on the good, and looking for social support. Thus, screen exposure here may reflect situational coping strategies rather than imitation of parental media use [19].

The weak and non-significant correlations between parental attitudes and self-efficacy and children’s screen use further illustrate the gap between beliefs and practices, indicating that although parents may hold certain negative beliefs and confidence regarding screen management, these do not necessarily translate into reduced screen time for children. Parents in the study often described screen time as inevitable, particularly in situations involving mealtime, tantrum control, or multitasking. While many held negative attitudes toward excessive screen use and expressed confidence in their ability to regulate it, they frequently conceded to its use due to pragmatic and situational demands. This finding aligns with previous research suggesting that although awareness of guidelines exists, their application is often perceived as impractical within the complexities of households [25, 31].

By contrast, the significant negative association between technology-related parenting and children’s screen time underscores the importance of deliberate mediation strategies. This finding is consistent with previous research indicating that active parental mediation, such as setting boundaries, supervising screen use, and removing devices from bedrooms, can effectively reduce children’s screen time [6, 13, 27, 30]. In contrast, passive parenting, characterized by a lack of supervision or engagement, has been associated with increased screen time in children [30, 32, 33]. The current findings reinforce the importance of controlling and rule-setting in managing children’s screen time.

### Strengths and limitations

The current study addresses a critical gap in the existing literature by examining the relationship between young children’s screen time and multiple parental factors, including parents’ own screen use, perceptions, and technology-related parenting within the Indian context. The use of validated measures and the consideration of underexplored variables such as technology-related parenting and parental self-efficacy add to the study’s contribution. Notably, the finding of only weak associations between parental and child screen time contrasts with previous research, highlighting the value of further investigation in this area.

However, several limitations must be acknowledged. The study used convenience sampling within a single hospital setting, which introduces sampling bias and may not reflect the broader population. The relatively small sample size (*n* = 123) limits statistical power and generalizability, especially given India’s large and diverse population. The cross-sectional design precludes any inference of causal relationships among the variables studied. Children’s screen time was parent-reported, raising the possibility of recall bias and social desirability bias, with potential for under- or over-reporting. Additionally, while validated measures were incorporated, the adaptation or local validity of these tools for the Indian context is not fully established. Factors like the number of children and working hours of parents were not taken into consideration, which may have influenced the relation between screen time. Finally, as many children in the sample were cared for by grandparents, not assessing grandparental screen habits or perceptions represents a further limitation.

Given these methodological constraints, the study offers valuable insights but does not provide a fully comprehensive framework. Future research should aim to address these limitations through larger, more representative samples, inclusion of all key caregivers, and longitudinal or mixed-methods designs.

### Clinical significance

Increasing screen time among young children is a growing concern due to its negative effects on various aspects of child development. Given that parents play a pivotal role in shaping children’s habits, growth, and development. This study highlights how parental behaviors, attitudes, and the establishment of rules and regulations around technology use are critical factors influencing children’s screen habits. By emphasizing these dimensions, the study lays essential groundwork for designing targeted interventions aimed at modifying parental practices, thereby contributing to the reduction of excessive screen time among young children.

### Future recommendations

Future research should aim to conduct more comprehensive and detailed investigations into the screen time behaviors of parents and children within Indian households in various socio-demographic backgrounds. Moreover, employing experimental or longitudinal designs would facilitate the determination of causal pathways and identify key factors that mediate or moderate this relationship, thereby informing the development of effective interventions to reduce excessive screen use among young children.

In addition, there is a notable gap in qualitative research within the Indian context that explores parental roles and perceptions in shaping children’s screen habits. Such studies would provide rich, contextualized insights into the complexities and cultural nuances that quantitative methods may overlook, deepening our understanding of the mechanisms underlying screen time behaviors. Finally, incorporating objective measurement tools, such as digital tracking technologies or time-use diaries, in future research will enhance the accuracy and reliability of screen time assessments, leading to more robust and generalizable findings.

## Conclusion

The aim of this research was to investigate the relationship between parents’ screen time and that of their young children. This study found only a weak and non-significant correlation between parents’ and young children’s screen time. Similarly, parental perceptions of screen time were not significantly related to children’s screen use. However, technology-related parenting, such as setting rules and monitoring, was linked to slightly lower screen time in young children. These findings highlight the nuanced and limited direct influence of parents’ own screen habits but emphasize the importance of active parenting strategies in managing children’s screen use within the Indian context. Future research should use longitudinal designs and larger, more diverse samples to better understand causal relationships and develop tailored interventions based on those results to promote healthy screen habits in children.

## Data Availability

The datasets used and analyzed during the current study are available from the corresponding author upon reasonable request.

## Abbreviations

AAP: American Academy of Pediatrics
WHO: World Health Organization
